# CO_2_ Laser Division of Neo-Vallecula Improves Dysphagia in the Postlaryngectomy Patient: A Case Series and Review of the Literature

**DOI:** 10.1155/2020/4015201

**Published:** 2020-10-19

**Authors:** Mohamad Z. Saltagi, Chelsey A. Wallace, Avinash V. Mantravadi, Michael W. Sim

**Affiliations:** ^1^Indiana University School of Medicine, Indianapolis, IN, USA; ^2^Department of Otolaryngology-Head and Neck Surgery, Indiana University School of Medicine, Indianapolis, IN, USA

## Abstract

**Objectives:**

To review the literature on neo-vallecula diagnosis and management and to report our findings regarding 3 patients who developed neo-vallecula in the context of free-flap pharyngeal reconstruction following total laryngectomy.

**Methods:**

This case series reports three patients who developed a neo-vallecula following a laryngectomy and free-flap pharyngeal reconstruction. All three patients were treated with a CO_2_ laser endoscopic procedure.

**Results:**

Neo-vallecula formation is thought to be related to tension on the neopharyngeal closure or closure technique following total laryngectomy. Diagnosis may be obtained with swallow studies, videofluoroscopy, or endoscopy. Treatment has included external excision and endoscopic procedures such as stapling, harmonic scalpel excision, and laser removal. We utilized an endoscopic approach entailing the use of a CO_2_ laser to divide the neo-vallecula, and all our patients reported improvement in their dysphagia.

**Conclusions:**

Treatment of an anterior neo-vallecula endoscopically using a CO_2_ laser is an effective way to treat dysphagia in patients following total laryngectomy with free-flap pharyngeal reconstruction.

## 1. Introduction

Patients with advanced laryngeal cancer are often treated with multiple modalities, including surgery and chemoradiation, to provide optimal survival outcomes [1]. While swallowing is sometimes an issue for patients after total laryngectomy, the cause of dysphagia can vary including salivary fistula, stricture, and/or pseudodiverticulum [1]. Various approaches have been used to evaluate and manage dysphagia in these contexts. We report a case series of three patients who developed dysphagia after total laryngectomy and free-flap reconstruction of their pharynx. Following initial diagnosis of malignancy, one patient was treated primarily with upfront surgery while the others underwent chemoradiation before surgery. Following laryngectomy and reconstructive procedures, these patients developed dysphagia due to a pseudodiverticulum formed at the tongue base, at the free-flap site. All three patients were treated with CO_2_ laser division of their pseudodiverticulum (hereby referred to as “neo-vallecula”).

## 2. Case Report: Patient 1

**Figure 1:**
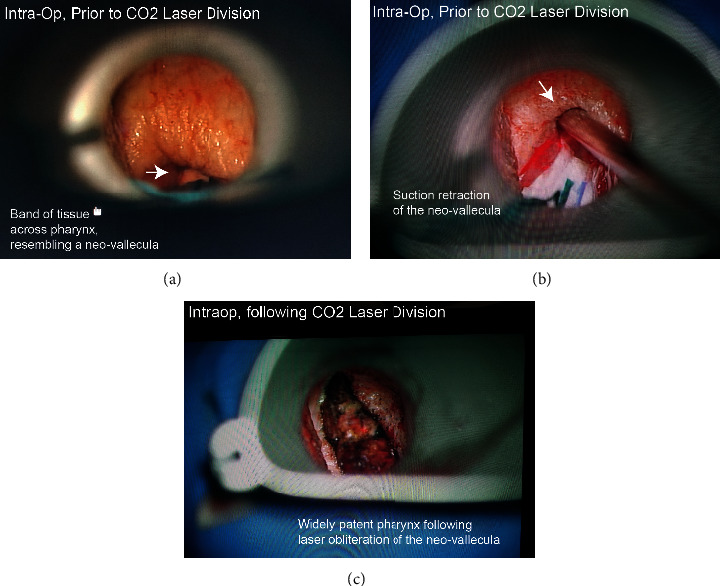
Intraoperative images: neo-vallecula before and after CO_2_ laser division. Images showing the intraoperative findings for patient 1. (a) A band of tissue, the neo-vallecula, is limiting a full view of the pharynx. (b) The band of tissue is retracted superiorly using a suction. (c) Intraoperative image of the band of tissue following CO_2_ laser division, demonstrating a widely patent pharynx.

A 63-year-old man presented to our clinic for evaluation of a large, obstructing supraglottic mass. Due to the risk of airway compromise, the patient underwent an awake tracheostomy and biopsy, which confirmed the diagnosis of locally advanced squamous cell carcinoma of the larynx. The patient then underwent a total laryngectomy and partial base of tongue resection, and reconstruction with an anterolateral thigh free flap to reconstruct the pharynx and anterior neck above the stoma. The patient completed chemoradiation about 5 months postoperatively, and during routine follow-up, was noted to have significant dysphagia clinically and on swallow study evaluation. The patient lected to undergo pharyngoscopy and CO_2_ laser diverticulectomy of his neo-vallecula. The diverticulectomy was conservative due to concern for pharyngocutaneous fistula formation. During initial follow-up, the patient noted some clinical improvements and was able to swallow liquids but remained unable to pass a pill through his neopharyngeal inlet. A second CO_2_ laser diverticulectomy was performed to remove the residual obstructing tissue, resulting in significant improvement, and the patient was able to tolerate a soft diet consistently thereafter. The intraoperative technique is shown in Figure 1.

## 3. Case Report: Patient 2

**Figure 2:**
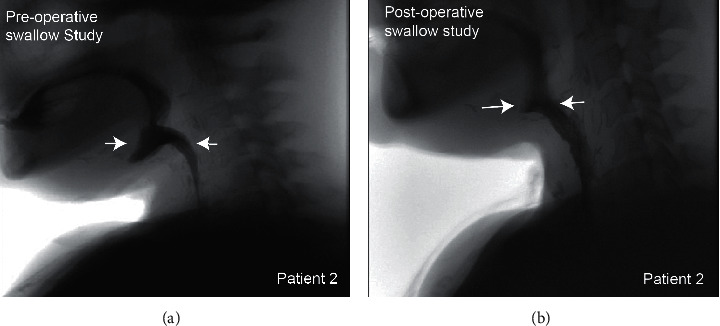
Patient 2 underwent preoperative and postoperative swallow studies. (a) A preoperative swallow study showing pooling of contrast within the neo-vallecular pouch (arrows). (b) A postoperative swallow study showing significant improvement, with most of the contrast making its way towards the esophageal inlet (arrows).

A 54-year-old woman presented to our clinic for evaluation of a supraglottic mass consistent with squamous cell carcinoma of the larynx. After treatment failure with chemotherapy and radiation, the patient underwent a salvage total laryngectomy and tongue base resection with a left forearm free-flap pharyngeal reconstruction. Following surgery, the patient was noted to have a pharyngocutaneous fistula, which ultimately required a pectoralis major flap. The fistula resolved, but during follow-up, the patient had persistent oropharyngeal dysphagia. Nasopharyngoscopy revealed that a neo-vallecula had developed within the patient's prior free-flap site, obstructing the pharyngeal lumen. The patient elected to undergo pharyngoscopy and CO_2_ laser diverticulectomy of the neo-vallecula. After surgery, a swallow study demonstrated absence of the shelf-like tissue protrusion seen on the preoperative study (Figure 2). Clinically, the patient's dysphagia improved and she was able to advance to a soft diet.

## 4. Case Report: Patient 3

A 47-year-old man presented for evaluation of a soft tissue lesion involving the left aryepiglottic fold, false and true vocal cords, with extension to the left pyriform sinus. The patient underwent laryngoscopy and biopsy confirming invasive moderately differentiated squamous cell carcinoma. Due to mass effect leading to obstruction of part of the airway, a laser was used to debulk some of the tumor, and then chemotherapy and radiation were initiated for definitive management. One year following treatment, direct laryngoscopy showed evidence of recurrence, and the patient underwent a total laryngectomy with primary closure and a right serratus overlay. Postoperatively, the patient had progressive dysphagia to solids. Laryngoscopy demonstrated a pharyngeal diverticulum at the tongue base, and the cervical esophagus was mildly stenotic. The patient underwent esophageal dilation using Maloney dilators and CO_2_ laser diverticulectomy of his neo-vallecula. Clinically, the patient's swallowing significantly improved, and he was able to tolerate a regular diet.

## 5. Discussion

Dysphagia is a relatively common complication following total laryngectomy and chemoradiation, with causes including neo-vallecula, pharyngocutaneous fistula, stricture, and reflux [1]. Following total laryngectomy and free-flap reconstruction, muscle contraction is limited at the suture lines within the operative site, reducing peristalsis during swallowing and often causing dysphagia [2]. Poor coordination of the remaining pharyngeal constrictor muscles can also contribute to dysphagia [3].

A neopharyngeal pseudodiverticulum or neo-vallecula is a common cause of dysphagia following total laryngectomy and free-flap reconstruction. Neo-valleculas are pouches located at the tongue base with a posterior band of tissue resembling a pseudoepiglottis, which obstructs the neopharyngeal lumen causing dysphagia [1]. Kirchner first reported neo-vallecula as a cause of dysphagia in 1962 [4]. In a study of 26 laryngectomy patients, Kirchner reported on 13 patients who had developed a pharyngocutaneous fistula, almost all of which were located where the pharyngeal suture lines met the base of the tongue [4] (Table 1). In patients whose fistulae did not reach the skin surface, a pouch or a neo-vallecula formed instead [4]. Of the 26 patients reported, 12 had dysphagia related to development of a neo-vallecula [4].

Clinically, patients with a neo-vallecula often present with regurgitation while swallowing and while there is food or mucus accumulation in the throat, sensation of a foreign body in the throat, and dysphagia. Kirchner hypothesized that breakdown of the pharyngeal closure due to tension on wound edges during swallowing, combined with contraction of tongue musculature, can lead to neo-vallecula development [4]. In this hypothesis, neo-vallecula development is related to the amount of tension created by the neopharyngeal closure [4].

Other studies have supported the claim that closure technique can impact neo-vallecula incidence. One group at VU University Medical Center of Amsterdam reported that in a retrospective cohort study of 117 laryngectomy patients, 84.6% of patients with a vertical closure developed a neo-vallecula versus 18.5% of patients with a T-shaped closure [9] (Table 1). Lippert et al. also noted correlation between neo-vallecula formation and neopharyngeal closure, with 67% of reported T-type closure patients and 100% of longitudinal closure patients developing a neo-vallecula [8] (Table 1). In contrast with the above, some studies have found no correlation between neo-vallecula and closure technique, pharyngocutaneous fistula incidence, or radiotherapy [6, 8].

In addition to hypothesizing causes of neo-vallecula formation, studies have investigated methods to diagnose this clinical pathology. Neo-valleculas are challenging to detect clinically, and use of barium swallow and endoscopic evaluation have helped facilitate diagnosis [2]. Transnasal flexible endoscopic examination followed with contrast radiography has been noted to be preferable to rigid pharyngoscopy, as the former has higher sensitivity for detecting neo-vallecula [14]. Despite multiple benefits, though, a disadvantage of using endoscopy is the inability to visualize oral and esophageal phases of swallowing, and this is an area where videofluoroscopy has a clear advantage [14]. All in all, endoscopic evaluation and videofluoroscopy are both useful tools in evaluating the patient with suspected neo-vallecula.

Various options have historically been pursued to manage neo-vallecula, including external and internal surgical interventions. External approaches, by design, require a neck incision to directly expose and eliminate the pouch [8]. Gacek described an external approach in which an esophagoscope is inserted to the depth of the neo-vallecula, and an anterior neck excision is made to meet the esophagoscope [10]. This incision is extended to transect the partition between the neo-vallecula and the esophagus, and scissors are used to resect the partition [10]. The final step is closure from the mucosal layers to the skin with interrupted sutures. Gacek used this method successfully in two patients: the first had “marked improvement in swallowing” and the second showed absence of neo-vallecula with barium study [10] (Table 1).

In contrast, internal approaches are less invasive and offer multiple options to address the neo-vallecula. One endoscopic option carried out by Deschler et al. entailed transoral use of monopolar cautery to perform a central wedge resection of tissue causing the neo-vallecula [11]. This method was performed on two patients with improvement in swallowing demonstrated by one patient's swallow study, and subjectively in the other patient [11]. This excision simply facilitated drainage and did not extend to the base of the neo-vallecula.

Another endoscopic option is endoscopic stapling [5]. D'Souza et al. noted endoscopic stapling had been previously used for posterior pharyngeal pouches and explored its use for an anterior pouch [5], reporting positive outcomes in one patient. A third internal approach is use of the harmonic scalpel; Jaber et al. used a harmonic scalpel to resect a neo-vallecula in one patient, as his team was intraoperatively unable to reach the distal pieces of the pouch with the endoscopic stapler [7]. Using the harmonic scalpel allowed his team to resect and simultaneously coagulate mucosal edges, and this patient tolerated a liquid diet at 2 months postoperatively with improved quality of life [7] (Table 1). Notably, this patient had not improved with serial dilations before the aforementioned surgery [7].

In addition to the above, the CO_2_ laser has been used as an internal approach to divide the excess tissue of a neo-vallecula [8]. Lippert et al. reviewed the treatment of 81 patients in Germany between 1984 and 1996 with a CO_2_ laser for dysphagia; of these patients, 11 had dysphagia from a neo-vallecula [8]. After treatment with the CO_2_ laser, 8 of 11 patients reported “entirely comfortable” swallowing without any remaining dysphagia symptoms, but 2 of these 8 required another operation within 9 months of the first due to dysphagia reoccurrence [8]. The remaining 3 patients reported “improved” swallowing but did not have complete resolution of their dysphagia [8] (Table 1). Another article described 9 patients treated with CO_2_ laser division of their neo-vallecula, reporting 8 of the 9 had “obvious improvement” in swallowing, with 7 patients additionally noting less food regurgitation and 5 noting less food accumulation [2] (Table 1). A third article used a CO_2_ laser in 4 patients, all with clinical resolution of symptoms and 2 with preoperative and postoperative swallow studies objectively demonstrating improvement [12] (Table 1). Overall, laser appears to have positive swallowing outcomes in this patient population. Advantages of low power CO_2_ laser include low bleeding and tissue trauma, low postoperative pain, and short hospitalization with quick recovery [8].

Our paper adds to the literature on the use of CO_2_ laser in the management of neo-vallecula. The strength of this paper lies in consistent operative technique (Figure 1) and our ability to obtain preoperative and postoperative swallow studies in some of our patients. We demonstrate improvement in dysphagia in patients with a neo-vallecula treated with CO_2_ laser division.

A limitation of this paper lies in the limited number of patients studied. However, the unique aspect of our cohort is that all patients had free-flap reconstruction following total laryngectomy, while much of the literature includes patients who underwent primary closure. Our patient selection process dictated that we limit the number of patients studied. Another limitation of this paper is that not all patients were able to obtain preoperative and postoperative swallow studies. However, we would argue that the clinical improvement in our patients' swallowing provides a more valuable marker of their improvement than swallow studies.

## 6. Conclusion

We report a case series of three patients who presented with dysphagia secondary to a neo-vallecula that developed following total laryngectomy and free-flap reconstruction. These patients were successfully treated with CO_2_ laser division, with notable clinical improvement in their swallowing. CO_2_ laser division of neo-vallecula is a minimally invasive, relatively safe, and adequate method to improve dysphagia caused by neo-vallecula formation following total laryngectomy and free-flap reconstruction.

## Figures and Tables

**Table 1:** Literature review summary table.

Article	Number of patients	Treatment offered	Swallowing outcomes	Complications
Endoscopic stapling of postlaryngectomy neopharyngeal anterior diverticulum [5]	1	Dilation of stricture (self-dilation with mercury bougies)	Worse	Enlarged the anterior neopharyngeal diverticulum
		Transoral endoscopic stapling of posterior wall of pouch	Patient stated swallowing was “best it had been since before laryngectomy”	None
Anterior diverticulum after total laryngectomy [6]	34	None (this article was a study to determine which types of laryngectomy lead to diverticulum formation)		

Postlaryngectomy dysphagia masking as velopharyngeal insufficiency: a simple solution for an anterior neopharyngeal diverticulum [7]	1	Serial dilations	No improvement	
		Transoral endoscopic stapling of nasopharyngeal diverticulum	Could not reach pouch	
		Harmonic scalpel to cleave distal portion of pouch	2-month follow-up: no significant regurgitation	None

Management of Zenker's diverticulum and postlaryngectomy pseudodiverticulum with the CO_2_ laser [8]	11	CO_2_ laser on tissue bridge	6 patients without swallowing difficulties after first operation, 2 patients required second operation and had no difficulties after operation, 3 patients had improved swallowing but not full resolution	One patient had parastomal fistula

Conservative management of a large postlaryngectomy neopharyngeal diverticulum [3]	1	Manual reduction of neck swelling	At 4-month follow-up, patient presented with dysphagia which was treated the same way	

Laser treatment of symptomatic anterior pharyngeal pouches after laryngectomy [2]	9	CO_2_ laser	8/9 noted significant improvement in swallowing (remaining patients still reported swallowing issues but had irradiation caries treated with full mouth extraction which could explain persistence of difficulties)	One patient had recurrence of neo-vallecula but reported no further issues after a second CO_2_ laser treatment. Another patient improved after operation but had recurrence of problems 6 months later

Influence of closure technique in total laryngectomy on the development of a pseudodiverticulum and dysphagia [9]		None (this was a review to determine correlation between closure and dysphagia and diverticulum formation)		
Swallowing after laryngectomy [1]		None (this is a review article)		

Management of vallecular pseudodiverticulum [10]	2	External approach (hypopharyngoscope could not reach inferior margin)	One patient had resolution of swallowing difficulties and the other had swallowing improvement	None reported

Disabilities resulting from healed salivary fistula [4]	12	None (review of cases, and in each, the diverticulum was simply diagnosed but not treated)		

Postlaryngectomy dysphagia caused by an anterior neopharyngeal diverticulum [11]	2	Transoral wedge resection	Resolution of swallowing difficulties	None

Postlaryngectomy neopharyngeal diverticula [12]	3	CO_2_ laser division	One required second procedure but had improvement of swallowing difficulties	None reported

Anterior neopharyngeal diverticulum following laryngectomy [13]	1	Endoscopic lysing of scar tissue	Relief of dysphagia	None reported
	1	External approach (transverse high cervical incision)	Complete resolution of swallowing problems	

The anatomy and complications of “T” versus vertical closure of the hypopharynx after laryngectomy (Davis)	5	Esophageal dilation	Improvement for 4/5	One patient needed laser excision and had improvement following procedure
